# ELP2‐NLRP3‐GSDMD/GSDME‐mediated pyroptosis is induced by TNF‐α in MC3T3‐E1 cells during osteogenic differentiation

**DOI:** 10.1111/jcmm.17994

**Published:** 2023-10-13

**Authors:** Changliang Xia, Shuanji Ou, Yang Yang, Wei Zhang, Wenjiao Wu, Qi Chen, Wenjun Li, Hanyu Lu, Yeyang Wang, Yong Qi, Changpeng Xu

**Affiliations:** ^1^ Department of Orthopaedics The Affiliated Guangdong Second Provincial General Hospital of Jinan University Guangzhou People's Republic of China

**Keywords:** bone infections, downstream pyroptosis, elongated protein 2, osteogenic differentiation, TNF‐α treatment

## Abstract

Tumour necrosis factor‐α (TNF‐α) is a cytokine involved in systemic inflammation. TNF‐α slows down osteogenic differentiation, which may contribute to poor bone development in the inflammatory microenvironment. TNF‐α inhibits osteogenic differentiation by activating the JAK‐STAT3 pathway, of which Signal transducer and activator of transcription 3 (STAT3)‐interacting protein 1 (StIP1, also known as elongator complex protein 2, ELP2) is a key protein in the JAK‐STAT3 pathway. We investigated whether and how ELP2 activation mediates the TNF‐α‐induced pyroptosis during osteoblastic differentiation. Using in vitro cell cultures of preosteoblastic MC3T3‐E1 cells, we found that TNF‐α exposure causes cell pyroptosis in an inflammatory microenvironment during osteoblastic differentiation. Bioinformatics, protein docking model and co‐immunoprecipitation analysis revealed an association between ELP2, STAT3 and NLRP3. Forced ELP2 expression promoted MC3T3‐E1 cells pyroptosis, with an increase in the expression of STAT3, NLRP3 inflammasome, GSDMD/GSDME, osteoblast marker genes, and the activity of alkaline phosphatase. In contrast, ELP2 silencing ameliorated MC3T3‐E1 cells pyroptosis, and osteogenic differentiation, especially after TNF‐α stimulation. The TNF‐α‐induced cells pyroptosis during osteoblastic differentiation was therefore mediated by ELP2. These results suggest that ELP2 is upregulated at the pyroptosis of MC3T3‐E1 cells and inhibits osteogenic differentiation in response to TNF‐α through NLRP3‐GSDMD/GSDME activation.

## 1 INTRODUCTION

Human bone mass is maintained through the balance between osteoblasts and osteoclasts, which play key roles in osteogenesis and bone resorption, respectively.^1^ Several chronic inflammatory conditions linked with bone formation, such as rheumatoid arthritis, systemic lupus erythematosus, and traumatic fractures, negatively affect bone mass and bone regeneration^2^, ^3^ by causing excessive cytokine secretion and inflammation that disrupts the equilibrium between bone production and resorption.^2^ Tumour necrosis factor (TNF‐α) is an important proinflammatory cytokine that affects inflammatory responses^4^ and has been shown to contribute toward the pathogenesis of osteomyelitis.^5^

TNF‐α induced inhibits osteogenic differentiation by downregulating the expression of osteoblast markers (including OCN, OPN and RUNX2) and alkaline phosphatase activity.^6^ Our earlier research used isobaric tags for relative and absolute quantitation (iTRAQ) combined with multiple reaction monitoring quantitative proteomics methods, revealed that ELP2 plays a key role in TNF‐α‐induced inhibition of osteoblast differentiation, which is associated with activation of transcriptional activator 3 (STAT3).^7^ ELP2 can regulate the ligand‐dependent activation of STAT3, also known as STAT3‐interacting protein 1 (StIP1). In addition, our further demonstrated that the TNF‐α‐induced inhibitory effect on osteoblastic differentiation was mediated by ELP2, which was linked with Janus kinase Janus kinase 2 (JAK2)/STAT3.^8^ The observations suggest that ELP2 acts downstream of TNF‐α to inhibit osteoblast differentiation.

In recent years, an increasing number of studies have linked inflammation to cell death and have demonstrated that cell death programs are dysregulated in various diseases, particularly those related to inflammation.^2^, ^9^ TNF‐α‐induced pyroptosis is associated with pathogenesis of inflammatory diseases. In non‐infectious diseases, such as cardiovascular disease, TNF‐α activates the innate immune system and NLRP3, which is subsequently assembled and further induces inflammatory cell death.^10^ In infectious diseases, such as influenza and severe acute respiratory syndrome coronavirus 2 (SARS‐CoV‐2), pyroptosis contributes to the resolution of respiratory viral infections and the pyroptosis‐induced dysregulation of inflammatory factors exacerbates immunopathology, thereby leading to tissue damage and excessive inflammation.^11^ Pyroptosis is a specific type of cell death that is thought to be associated with inflammation^12^ and is typically characterized by cell swelling and membrane rupture regulated by members of the gasdermin protein family.^13^ For example, GSDMD is cleaved by inflammasome‐activated caspase‐1 to generate N‐terminal fragments that cause pyroptosis,^14^ while the activation of caspase‐3 results in GSDME cleavage.^15^ Although the discovery of pyroptosis has provided a potential new strategy for treating inflammatory conditions, such as bone inflammation, the specific molecular mechanisms have not yet been elucidated. The NOD‐like receptor thermal protein domain associated protein 3 (NLRP3) inflammasome activation is a potential molecular mechanism underlying pyroptosis that is thought to affect various diseases, including osteomyelitis, Alzheimer's disease and Type II diabetes.^16^, ^17^, ^18^, ^19^

MC3T3‐E1 has been used to explore osteoblast differentiation.^20^, ^21^, ^22^ Based on the results of our previous studies, we hypothesized that ELP2 promotes the deleterious effects of TNF‐α‐induced pyroptosis during osteoblastic differentiation in MC3T3‐E1 cells. TNF‐α induced a pyroptosis morphology in MC3T3‐E1 cells, which was further confirmed by Western blot analysis showing an increase in the expression of pyroptosis execution proteins. The involvement of ELP2 in the regulation of MC3T3‐E1 pyroptosis was then demonstrated through gene manipulation experiments. Subsequently, we employed bioinformatics analysis, protein docking modelling, and co‐immunoprecipitation to uncover an association between ELP2, STAT3, and NLRP3. Our findings suggest that the newly discovered role of ELP2 may hold promise for developing novel therapeutic strategies to mitigate the detrimental effects of excessive inflammation in various human diseases.

## 2 MATERIALS AND METHODS

### 2.1 Reagents and antibodies

Minimum essential medium α (MEM‐α) was obtained from Gibco/Invitrogen (catalogue no. 12571‐063; Grand Island, NY, USA). Fetal bovine serum was purchased from HyClone Laboratories (Logan, UT, USA). L‐ascorbate and β‐glycerophosphate were purchased from Sigma Chemicals (St. Louis, MO, USA). Trypsin was obtained from Gibco. Recombinant mouse TNF‐α (aa 80‐235) protein was purchased from R&D Systems (catalogue no. 410‐MT‐010 Shanghai, China). Lactate dehydrogenase (LDH) kits were purchased from Beyotime (Haimen, China). Alkaline Phosphatase Live Stain, SYTOX Green, and TRIzol™ reagents were purchased from Thermo Fisher Scientific (Waltham, MA, USA). Primary and secondary antibody diluents for Western blot (WB) analysis, COIP kits, and 2 × RealStar Fast SYBR qPCR kits were purchased from Absin (Shanghai, China). Bicinchoninic acid (BCA) protein assay kits were purchased from Takara (Dalian, China). ELP2 antibodies (NBP2‐16321) were purchased from Novus Biologicals (CO, USA). Cleaved caspase‐3 (9661S), caspase‐3 (9662S), cleaved caspase‐1 (Asp296), IL‐1β (12242S), RUNX2 (12556S), Asc (67824S), IgG control (3900S), and Gapdh (2118S) antibodies were purchased from Cell Signaling Technology (Boston, MA, USA). GSDMD (ab209845), anti‐DFNA5/GSDME (ab215191), caspase‐1, IL‐18 (ab71495), and NLRP3 (ab263899), and Alp (ab229126) antibodies were purchased from Abcam (Cambridge, MA, USA). Flag (20543‐1‐AP) antibodies were purchased from Proteintech (Chicago, IL, USA). ELP2 small guide RNA (sgRNA) vectors and GV341 expression vectors were constructed by Genechem (Shanghai, China). IL‐1β, IL‐18, OPN, OCN, RUNX2, and BMP2 ELISA kits constructed by Sembega (Nanjing, China) were used in the present study.

### 2.2 Cell culture and stimulation

The murine preosteoblast MC3T3‐E1 clonal cell line was purchased from iCell Bioscience (Shanghai, China). Plasmid constructs and cell transfection were performed as described previously.^23^, ^24^ In brief, the full‐length nucleotide sequence of mouse ELP2 (NCBI: NM_021448.2) was amplified using real‐time polymerase chain reaction (RT‐PCR; Table 1) and cloned into the GV341 expression vector to construct the recombinant Flag‐GV341‐ELP2 vector. Cells were then transfected with either Flag‐GV341‐ELP2 to overexpress ELP2 or a blank GV341 vector using a lentivirus according to the manufacturer's protocol. The cells were then transfected with either the Lenti‐sgRNA‐CAS9 virus or the control sgRNA vector for 3 days according to the manufacturer's instructions. Transfected cells were screened using puromycin to select the monoclonal sgRNA‐knockdown strain for purification. For further experiments, cells were plated in MEM‐α supplemented with 10% fetal bovine serum (FBS) at 37°C in a humidified atmosphere with 5% CO_2_. Trypsinized MC3T3‐E1 cells were seeded into six‐well plates at a density of 2 × 10^5^ cells/mL and cultured in a differentiation induction medium. The samples were prepared in triplicates and the high‐quality samples were used in further experiments. When the MC3T3‐E1 cells reached 70% confluence, they were cultured in osteoblast differentiation medium (MEM‐α containing 10% FBS, 0.1 mM dexamethasone, 10 mM b‐glycerol phosphate, 50 mM ascorbic acid) for 0–14 days.^6^ The culture medium was replaced once every 2 days with 2 mL of fresh medium. Fourteen days later, the cells were analysed and the following experiment was performed.

**TABLE 1:** Primers/overexpression and RNA‐interference sequences used in this study.

Name	Sequence	
ELP2	5’‐GCCATGAAGGACCTGTTTGT‐3′(forward)	For qPCR
	5’‐GCCAGGCAGACAGAAAGAAC‐3′(reverse)	
Runx2	5’‐GCTGTTAACTTCAAGTCCCT‐3′(forward)	
	5’‐GAAATCAAGTTCGAGGAAGC‐3′(reverse)	
IL‐18	5′‐TGCATCAACTTTGTGGCAAT‐3′(forward)	
	5′‐ATAGAGGCCGATTTCCTTGG‐3′(reverse)	
IL‐1β	5′‐TGTGGCAGCTACCTATGTCT‐3′(forward)	
	5′‐GGGAACATCACACACTAGCA‐3′(reverse)	
OPN	5’‐GCTGAATTCTGAGGGACTAAC‐3′(forward)	
	5’‐CTGTAAAGCTTCTTCTCCTCTG‐3′(reverse)	
OCN	5’‐AGCACCAGAATCTATCTGAA‐3′(forward)	
	5’‐AATGCCTTGTTCTCCTCTTA‐3(reverse)	
GAPDH	5’‐CCTTCCGTGTTCCTACCC‐3′ (forward)	
	5’‐CAACCTGGTCCTCAGTGTAG‐3′ (reverse)	
pcDNA3‐ELP2	5′‐CTGGGATCCATGGTTTCTTCTGTGCTG‐3′ (forward)	For overexpression
	5′‐CTGCTCGAGTCACAGTGCGCGTCTGTTAAC‐3′ (reverse)	
sgRNA ELP2 sgRNA NC	AGCGGTGTGGCCATTCAGAT	For knockdown
	CGCTTCCGCGGCCCGTTCAA	For control

Abbreviations: qRT‐PCR, quantitative real‐time polymerase chain reaction; sgRNA, small guide RNA.

### 2.3 Optical microscopy and scanning electron microscopy (SEM)

Trypsinized MC3T3‐E1 cells were seeded into six‐well plates at a density of 2 × 10^5^/mL in differentiation induction medium. The cells were then stimulated with or without 50 ng/mL TNF‐α for 24 h, fixed with paraformaldehyde for 10 min, washed twice with phosphate buffer saline, and imaged using an optical microscope. The number of dead cells was counted using a cell counter. MC3T3‐E1 cells were then reactivated and adjusted to a density of 2 × 10^5^/mL in a six‐well plate containing 25 mm^2^ cell slides in each well. Cultured cells were then exposed to TNF‐α for 24 h in an incubator, washed twice with 2 mL PBS, incubated with 2.5% glutaraldehyde (3 mL) at room temperature (25°C) for 1 h, and stored at 4°C.

SEM samples were cultured in six‐well plates containing 25 mm^2^ cell slides using a conventional incubator (37°C and 5% CO_2_). After exposure to TNF‐α for 24 h, cells were fixed with 2.5% glutaraldehyde, stored at 4°C overnight, dehydrated, electroplated, and observed using SEM (Hitachi S3400N, Tokyo, Japan).

### 2.4 Quantitative real‐time PCR

Total RNA was extracted from cells using TRIzol, purified using CHCl3 and ethanol, and equilibrated using a Nanodrop spectrophotometer (Pultton P100+ type). RNA was reverse transcribed into cDNA using an miDETECT A Track miRNA RT‐qPCR Starter Kit according to the manufacturer's instructions and using the primers listed in Table 1. Next, RT‐qPCR was performed using a 2 × RealStar Fast SYBR qPCR kit on a Roche Lightcycler480 (Switzerland, Basel) system according to the manufacturer's instructions. Data were compared using the 2^−ΔΔCt^ method.

### 2.5 Immunoblot analysis

Extracted proteins were quantified using a BCA kit at a wavelength of 562 nm. Next, 12%–15% sodium dodecyl sulfate‐polyacrylamide gel electrophoresis (SDS‐PAGE) was performed using 5× loading buffer in a metal bath for 10 min. Separated proteins were transferred to NC membranes, incubated with high efficiency blocking solution for 30 min, and incubated with primary antibodies in a shaker at 4°C overnight. After three washes (10 min each), the membranes were incubated with secondary antibodies at room temperature for 2 h, washed four times, and target bands were visualized using an Odyssey^®^ CLx Imaging System (LI‐COR, USA). The results were quantitatively analysed using ImageJ software.

### 2.6 Cytotoxicity and cytokine assay

LDH assays were performed according to the manufacturer's instructions and a standard curve was prepared using bovine heart lactate dehydrogenase. After TNF‐α stimulation, MC3T3‐E1 cell death was estimated by measuring LDH release at 450 nm using a microplate reader (Thermo Fisher).

Osteoblast differentiation and function is influenced by OCN and OPN, via interaction with osteoblast surface receptors and signalling pathways. RUNX2 acts as a transcriptional regulator, controlling the expression of OCN, OPN, BMP2 and other bone‐related genes. Together, these proteins contribute to the maintenance of bone integrity and play crucial roles in osteogenic differentiation. IL‐1β, IL‐18, OPN, OCN, RUNX2 and BMP2 assays were performed using the according to the manufacturer's instructions. Absorbance was measured at 450 nm using a microplate reader (Thermo Fisher).

### 2.7 Alkaline phosphatase (ALP) staining

After differentiation and cell stimulation, ALP staining was performed on cells in six‐well plates. Alkaline phosphatase was determined by measuring the conversion of p‐nitrophenyl phosphate to p‐nitrophenol. Briefly, cells were washed with PBS twice, fixed with 4% paraformaldehyde for 30 min at 37°C. Next, the cells were washed with TBST twice, and stained with 5‐bromo‐4‐chloro‐3‐indolyl phosphate plus nitroblue tetrazolium chloride in Tris–HCl, NaOH and MgCl_2_, successively, incubated with ALP working solution (2 mL) in tin foil in an incubator overnight, and imaged with a unified background. The number of stained cells were analysed using ImageJ software.

### 2.8 Fluorescence imaging

Cell viability was determined using SYTOX® Red staining, according to the manufacturer's instructions. Briefly, cells were stimulated and washed twice with PBS. Next, cells were stained with SYTOX^®^ Red stain (1 mL for per well of six‐well plate) for 15 min at 37°C and 5% CO_2_. Subsequently, brightfield and fluorescence images were captured using a Leica (Shanghai, China) confocal microscope with excitation and emission wavelengths of 633–635 and 660 nm, respectively.

### 2.9 Flow cytometry

Cells harvested using Trypsin–EDTA (0.25%) were adjusted to a concentration of 1 × 10^5^ to 5 × 10^7^ cells/mL using PBS. A 1 mL aliquot of the cell suspension was passed through a flow cytometry tube with a strainer and then 1 μL of SYTOX^®^ Red stain (Component A) was added to each tube at a final concentration of 5 nM and incubated for 15 min at room temperature (26°C) or 2–6°C in the dark. Samples were then analysed immediately, using excitation and emission wavelengths of 633–635 nm and 660 nm, respectively.

### 2.10 Immunoprecipitation

Briefly, MC3T3‐E1 cells with high ELP2 expression were lysed using high‐efficiency RIPA lysate in protease phosphatase‐inhibited buffer. After centrifugation at 16,000 × g for 10 min, the lysates were incubated with either IgG control or Flag antibodies and protein A/G PLUS‐Agarose overnight at 4°C. After five washes with TBST buffer, immunoprecipitated samples were added to 1× SDS loading buffer and collected after denaturation in a metal bath at 100°C.

### 2.11 Binding site prediction

Computerized virtual prediction technique is used for simulating protein molecular interactions and predicting binding sites^25^ and has been applied to predict protein interactions.^26^, ^27^ According to previous studies, potential STAT3 binding sites in selected human and mouse genes were predicted using the position weight matrix (PWM) algorithm from TRANSFAC to identify promoter regions, which were defined from the transcriptional start site.

### 2.12 Statistical analysis

Data were analysed using the Statistical Package for the Social Sciences (SPSS; SPSS Inc., Chicago, IL, USA) version 11.0 for Windows. Normally distributed data from different groups were compared using *t*‐tests, with *p* < 0.05 considered statistically significant. Data were visualized using GraphPad 9 (GraphPad Prism, San Diego, CA, USA) and Adobe Photoshop 2020 (Adobe Systems, San Jose, USA).

## 3 RESULTS

### 3.1 TNF‐α exposure causes MC3T3‐E1 cell pyroptosis via ELP2

**FIGURE 1:**
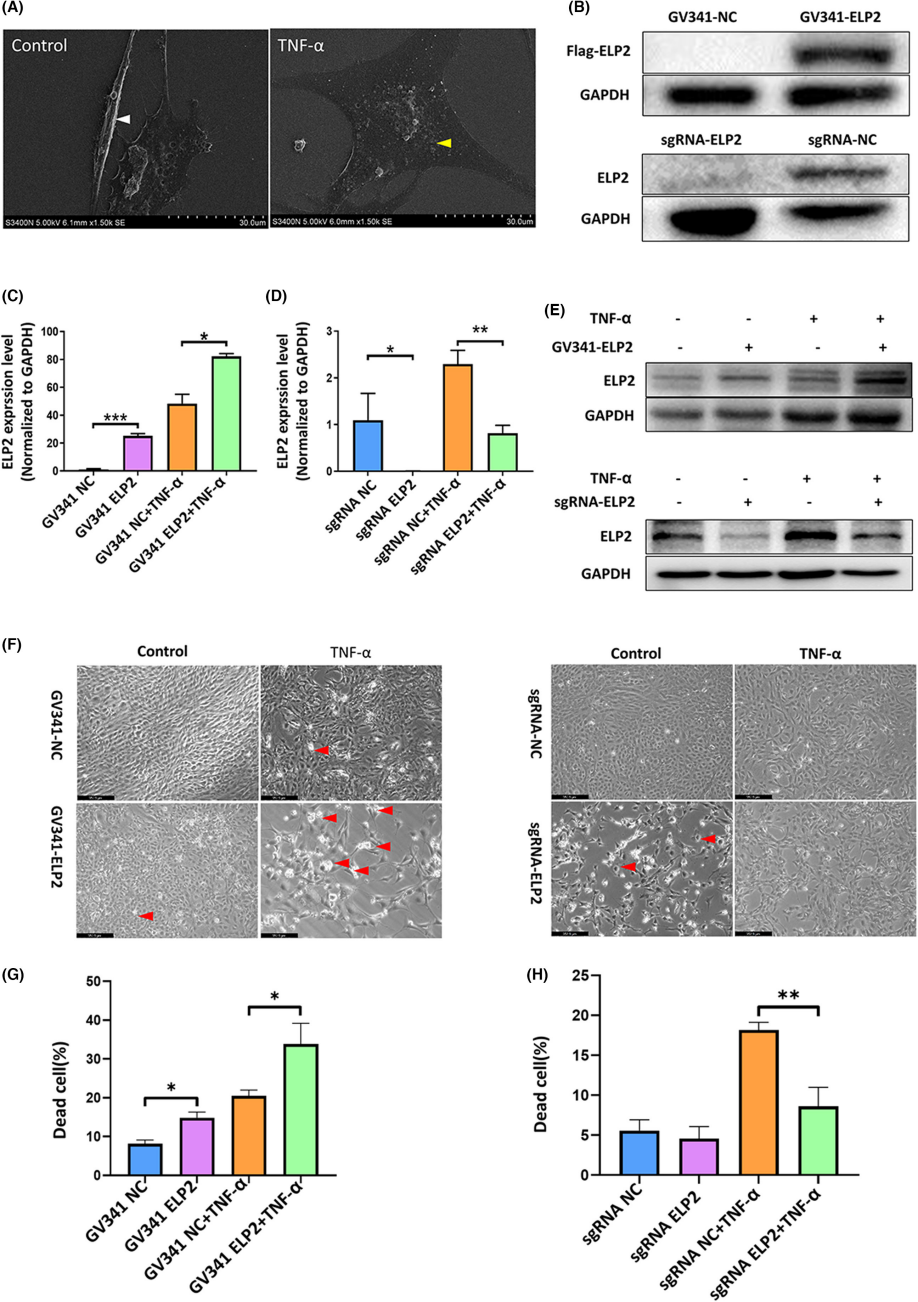
TNF‐α exposure causes MC3T3‐E1 cell pyroptosis via ELP2. (A) Scanning electron microscope (SEM) (5000× magnification, scale bar = 30 μm) images of cell morphology; TNF‐α (50 ng/mL) stimulation for 24 h cause flattening death of MC3T3‐E1 cells and display a “fried egg”‐like morphology. (yellow arrows represent pyroptosis cells; white arrows represent NC cells). (B) Effects of ELP2 vector construction, transfection, and knock‐down. ELP2 was successfully up‐regulated (GV341‐ELP2) or knocked down (sgRNA‐ELP2). (C, D, E) Results of western blot and quantitative real‐time PCR analysis showed that ELP2 overexpression (GV341‐ELP2) significantly increased the elevation in ELP2 induced by TNF‐α (50 ng/mL 24 h); while ELP2 knock down (sgRNA‐ELP2) reduced the elevation in ELP2 induced by TNF‐α (50 ng/mL 24 hr). (F) Light microscopic (50 × magnification) images of cell morphology. (red arrows represent death cells) Images show that ELP2 overexpression (GV341‐ELP2) significantly increased the cells death and Cell morphology abnormalities induced by TNF‐α (50 ng/mL 24 h), while ELP2 knock down (sgRNA‐ELP2) reduced the cells death and cell morphology abnormalities induced by TNF‐α (50 ng/mL 24 h). (G, H) Quantitative analysis of the dead cells revealed the rate of cell death between NC and ELP2 overexpression cells. Error bars represent the mean ± SD, and comparisons were performed using one‐way anova. *p > 0.05, *p < 0.05, **p < 0.01 and ***p < 0.001. *n* = 3 in each group. TNF‐α, tumour necrosis factorα; ELP2, elongator complex protein; PCR, polymerase chain reaction; sgRNA, small guide RNA; SD, standard deviation.

First, we detected morphological changes in MC3T3‐E1 cells treated with and without TNF‐α for 24 h using optical microscopy and SEM. Normal control (NC) cells were generally spindle‐shaped with an intact cell membrane (Figure 1A); then, cell morphology became indeterminate after TNF‐α exposure due to severe cell membrane damage and the cells died after releasing inflammatory factors (Figure 1F). A higher mortality rate was observed in TNF‐α‐stimulated or ELP2 overexpression samples (Figure 1G,H). In particular, the TNF‐α‐treated and overexpressed ELP2 cells displayed a ‘fried egg’‐like morphology, with swollen and flattened cytoplasm, pyroptotic bodies, and condensed chromatin (Figure 1A).

WB and RT‐qPCR analyses confirmed successful ELP2 overexpression following cellular transfection with the constructed pcDNA‐ELP2‐Flag vector (Figure 1B–D). In addition, stable low‐ELP2 expressing cell lines were obtained using CAS9 lentiviral particle transfection, screening, and clone selection (Figure 1B–D). Interestingly, we found that TNF‐α activated ELP2 expression and that TNF‐α knockdown significantly inhibited ELP2 expression (Figure 1E).

### 3.2 TNF‐α exposure leads to MC3T3‐E1 cell inflammation by activating the ELP2/NLRP3 pathway

**FIGURE 2:**
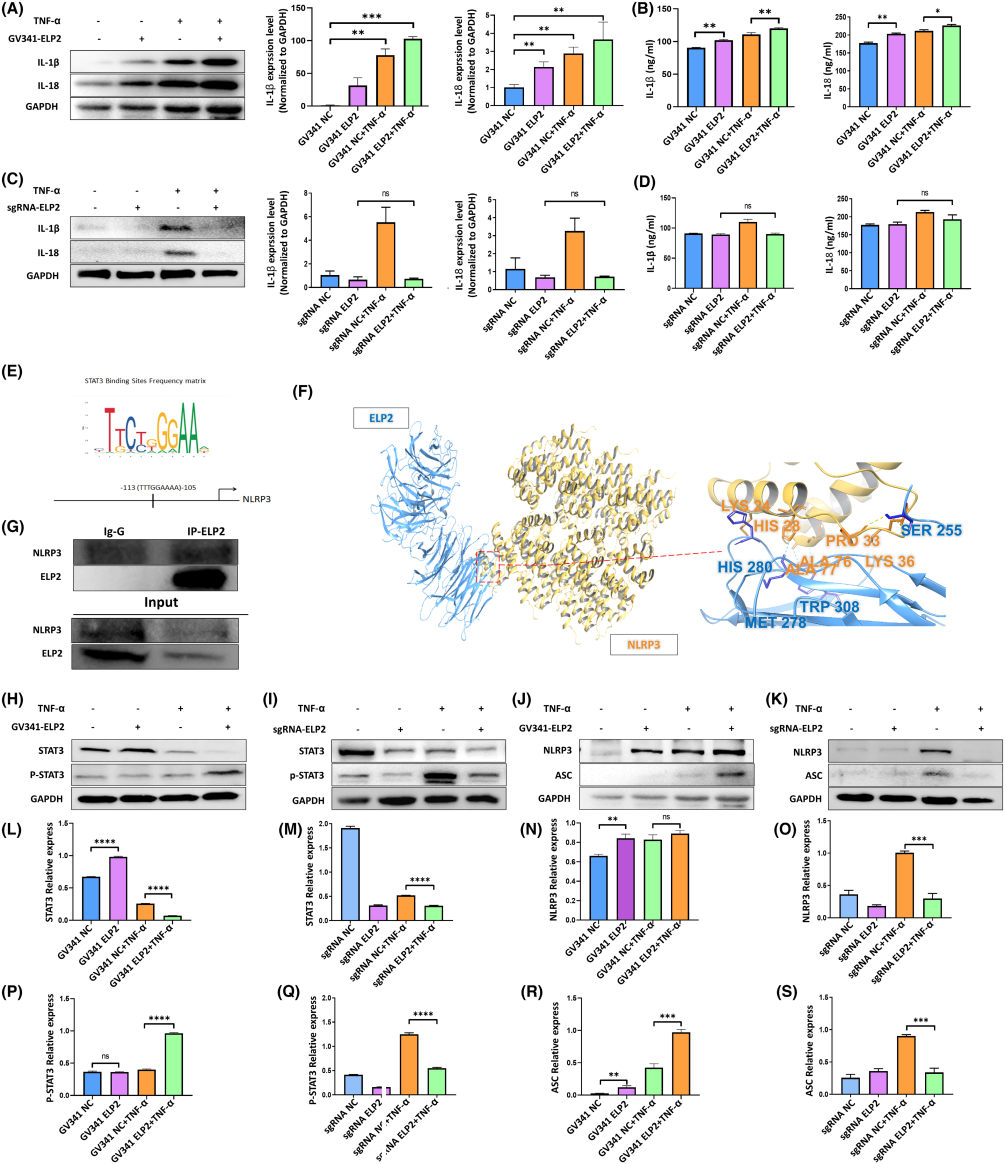
TNF‐α exposure leads to MC3T3‐E1 cell inflammation through ELP2/NLRP3 pathway. Western blot, quantitative real‐time PCR, and Elisa analysis showed that (A, B) ELP2 overexpression (GV341‐ELP2) significantly increased the elevation in IL‐1β and IL‐18 induced by TNF‐α (50 ng/mL 24 h); (C, D) and ELP2 knock down (sgRNA‐ELP2) reduced the elevation in IL‐1β and IL‐18 induced by TNF‐α (50 ng/mL 24 h). (E) Depiction of the putative NLRP3 binding site −115/−105 (ELP2) bp upstream of the transcription initiation site in the human STAT3 promoter and (F) Prediction of possible combinations 147 of NLRP3 and ELP2; the results of the interaction bridge analysis indicate that ELP2 can maintain binding by generating multiple non‐covalent bonds with amino acids within the NLRP3 pocket. (G) Results of immunoprecipitation analyses show interaction of ELP2 with NLRP3. (H) Western blot analysis and (L, P) quantitative PCR analysis results showed that ELP2 overexpression (GV341‐ELP2) significantly increased the elevation in phosphorylation of STAT3 induced by TNF‐α (50 ng/mL 24 h); (I, M, Q) ELP2 knock down (sgRNA‐ELP2) reduced the elevation in phosphorylation of STAT3 induced by TNF‐α (50 ng/mL 24 h). (J) Western blot and (N, R) quantitative PCR analysis showed that ELP2 overexpression (GV341‐ELP2) significantly increased the elevation in NLRP3 and ASC induced by TNF‐α (50 ng/mL 24 h); (K, O, S) ELP2 knock down (sgRNA‐ELP2) reduced the elevation in NLRP3 and ASC induced by TNF‐α (50 ng/mL 24 h). Error bars represent the mean ± SD, and comparisons were performed using one‐way anova. *p > 0.05, *p < 0.05, **p < 0.01 and ***p < 0.001. *n* = 3 in each group. TNF‐α, tumour necrosis factorα; ELP2, elongator complex protein; PCR, polymerase chain reaction; sgRNA, small guide RNA; STAT3, signal tran sducer and activator of transcription 3; NLRP3, NOD‐like receptor thermal protein domain associated protein 3; ASC, apoptosis‐associated speck‐like protein containing a CARD; SD, standard deviation.

Since pyroptosis is usually accompanied by the release of inflammatory factors, we measured IL‐1β and IL‐18 release to detect cellular inflammation. TNF‐α stimulation for 24 h (50 ng/mL) significantly increased IL‐1β and IL‐18 protein and mRNA levels (Figure 2A) compared to NC cells (TNF‐α, 0 ng/mL), with similar changes in mRNA and protein levels. In contrast, knockdown of ELP2 successfully blocked the release of inflammatory factors (Figure 2C). Moreover, these results were confirmed by measuring the release of inflammatory factors in the supernatant (Figure 2B,D).

Next, we used bioinformatics to predict the presence of an element in the promoter region (−113/−105 bp) of the human NLRP3 gene (Figure 2E) and found that this might be associated with elevated STAT3 expression. ELP2 is an important ligand during STAT3 activation, so we further explore the binding of ELP2 to NLRP3. The ZDOCK Score values 132 and their best pose interaction were calculated. The ZDOCK Score of NLRP3 and Elp2 133 was 1316.188. NLRP3 forms multiple non‐covalent bonds links with amino acid sites such as LYS24‐ HIS280, HIS28‐ MET278. As show proteins NLRP3 and Elp2 formed a stable protein docking model 134 (Figure 2F). In addition, we predicted that ELP2 may be associated with NLRP3. Coimmunoprecipitation assays with Flag antibodies in ELP2‐overexpressing cells confirmed the immunoprecipitation of ELP2 and NLRP3 (Figure 2G), suggesting that ELP2 strongly interacts with NLRP3. To determine the specific mechanism through which TNF‐α causes inflammation, we detected inflammasome activation and STAT3 phosphorylation. Under TNF‐α stimulation, STAT3 phosphorylation was clearly elevated (Figure 2H) and this increase was more pronounced during ELP2 overexpression (Figure 2H,L,P). As expected, ELP2 knockdown significantly reduced STAT3 phosphorylation compared to NC cells (Figure 2I,M,Q). After TNF‐α exposure for 24 h, NLRP3 and ASC protein expression increased significantly compared to NC cells (Figure 2J,N,R), suggesting that TNF‐α leads to MC3T3‐E1 cell inflammation via the ELP2/NLRP3 pathway. Moreover, ELP2 overexpression significantly increased NLRP3 and ASC protein expression levels, whereas knocking down ELP2 prevented NLRP3 and ASC expression (Figure 2K,O,S).

### 3.3 Regulatory targets of ELP2 affect pyroptosis caused by GSDMD/GSDME

**FIGURE 3:**
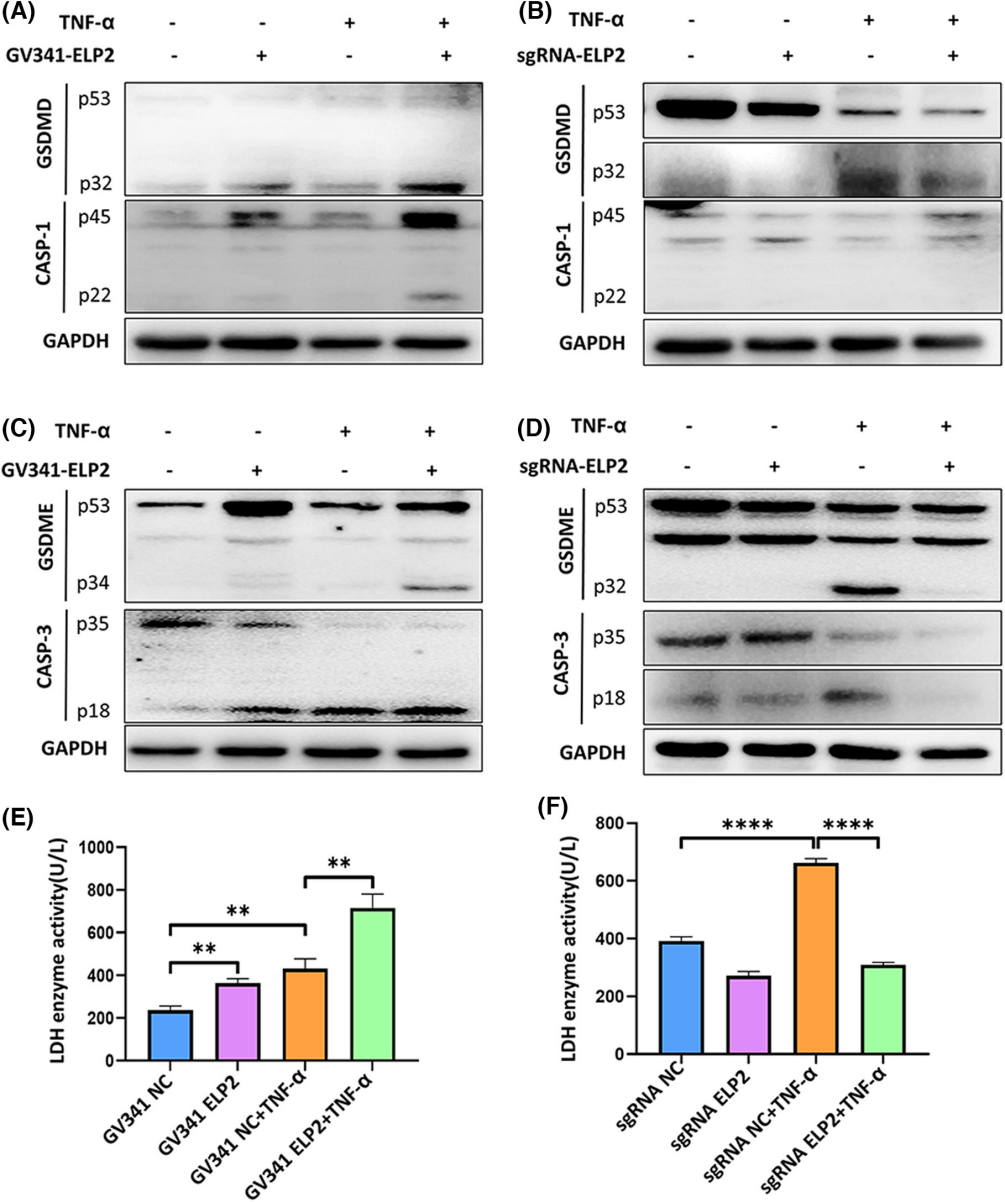
Regulatory targets of ELP2 affect pyroptosis caused by GSDMD/GSDME. A Western blot analysis showed that ELP2 overexpression (GV341‐ELP2) significantly increased the elevation in GSDMD‐N(p32) and cleave‐caspase1(p20) induced by TNF‐α (50 ng/mL 24 h); (B) ELP2 knock down (sgRNA‐ELP2) reduced the elevation in GSDMD‐N(p32) and cleave‐caspase‐1 (p22) induced by TNF‐α (50 ng/mL 24 h). (C) Western blot analysis showed that ELP2 overexpress (GV341‐ELP2) significantly increased the elevation in GSDME‐N (p34) and cleave‐caspase‐3 (p18) induced by TNF‐α (50 ng/mL 24 h); (D) ELP2 knock down (sgRNA‐ELP2) reduced the elevation in GSDMD‐N (p32) and cleave‐caspase1 (p20) induced by TNF‐α (50 ng/mL 24 h). (E) LDH release analyses showed that ELP2 overexpression (GV341‐ELP2) significantly increased the elevation in LDH level induced by TNF‐α (50 ng/mL 24 h); (F) ELP2 knock down (sgRNA‐ELP2) reduced the elevation in LDH level induced by TNF‐α (50 ng/mL 24 h). Error bars represent the mean ± SD, and comparisons were performed using one‐way anova. *p > 0.05, *p < 0.05, **p < 0.01 and ***p < 0.001. *n* = 3 in each group. TNF‐α, tumour necrosis factorα; ELP2, elongator complex protein; sgRNA, small guide RNA; LDH, lactate dehydrogenase; GSDMD, Gasdermin D; GSMDE, Gasdermin E; SD, standard deviation.

To determine whether TNF‐α inflammasome activation through the STAT3/NLRP3 pathway can cause cell pyroptosis by opening GSDMD/GSDME membrane pores, which have been reported to alter membrane permeability and induce pyroptosis,^28^, ^29^, ^30^ we measured the expression levels of GSDMD/GSDME and its associated proteins. Interestingly, we detected GSDMD/GSDME cleavage in MC3T3‐E1 cells exposed to TNF‐α (50 ng/mL) for 24 h, which led to pyroptosis (Figure 3). This may be due to GSDMD/GSDME cleavage and membrane pore activation by cleaved caspase‐1 and caspase‐3 (Figure 3A,C). Notably, GSDMD/GSDME protein activation increased significantly in ELP2‐overexpressing cells stimulated with TNF‐α, whereas no clear GSDMD/GSDME activation was observed after ELP2 knockdown, even with TNF‐α stimulation (Figure 3B,D). These findings suggest that ELP2 plays an important role in TNF‐α‐mediated inflammasome activation leading to pyroptosis. Therefore, we measured LDH activity to evaluate cell death (Figure 3E,F), finding that LDH release was consistent with the observed patterns of GSDMD/GSDME protein expression.

**FIGURE 4:**
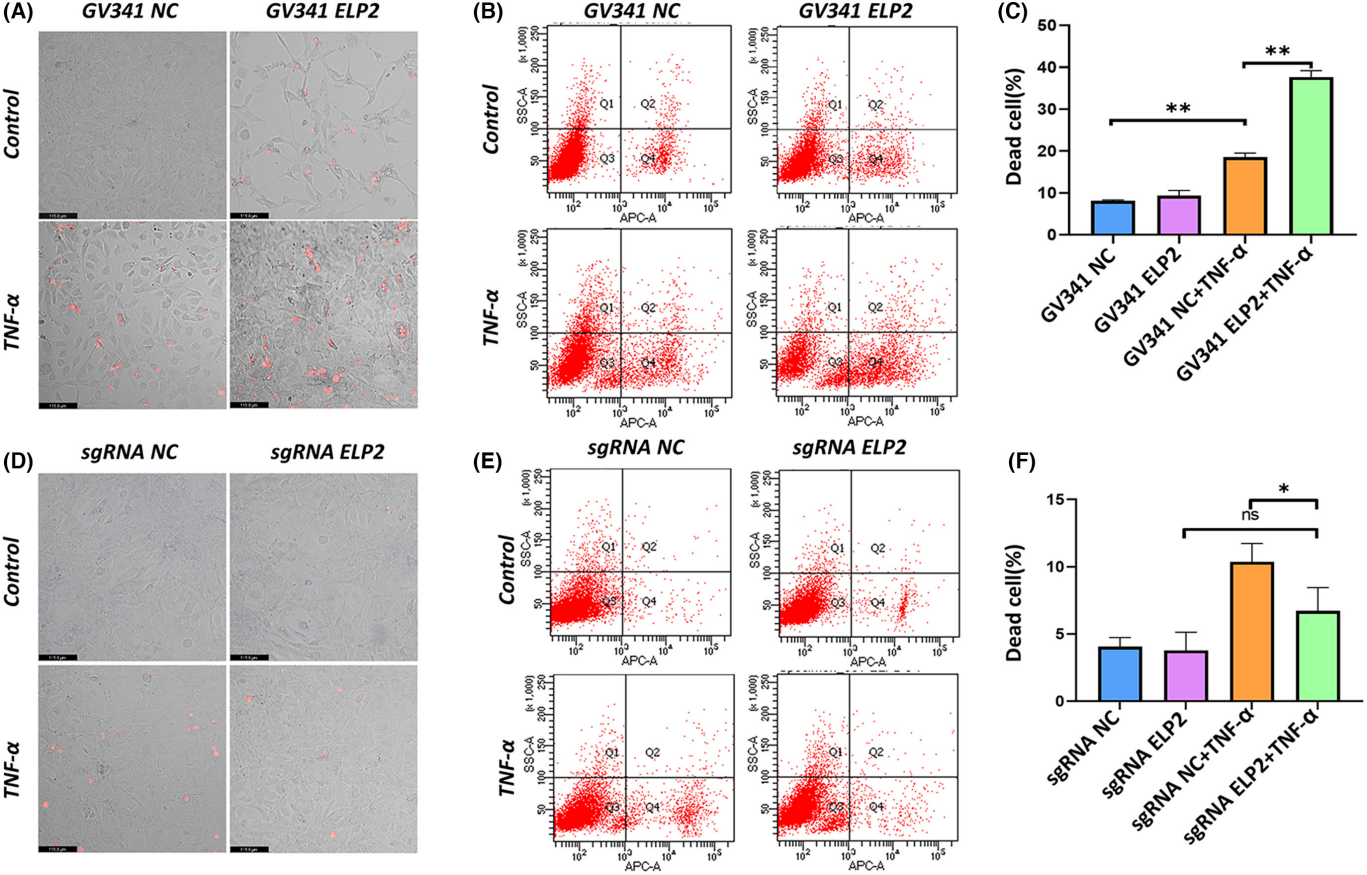
Effects of ELP2 on pyroptosis caused by GSDMD/GSDME. (A–C) Fluorescent staining and quantitative flow analysis show that ELP2 overexpression (GV341‐ELP2) significantly increased the cells death induced by TNF‐α (50 ng/mL 24 h); (D–F) ELP2 knock down (sgRNA‐ELP2) reduced the cells death induced by TNF‐α (50 ng/mL 24 h). Live cells (Q3) were distinguished from dead cells. Error bars represent the mean ± SD, and comparisons were performed using one‐way anova. *p > 0.05, *p < 0.05, **p < 0.01 and ***p < 0.001. *n* = 3 in each group. TNF‐α, tumour necrosis factorα; ELP2, elongator complex protein; sgRNA, small guide RNA; SD, standard deviation.

To evaluate the degree in which ELP2 affects pyroptosis, we quantitatively measured cell death using fluorescent staining and flow cytometry. After 24 h of TNF‐α treatment, cells showed a significant increase in necrotic area compared to the NC group (*p* < 0.05; Figure 4). In addition, TNF‐α caused massive pyroptosis after ELP2 overexpression compared to the NC group (Figure 4A–C), whereas cell death was decreased significantly after ELP2 knockdown (Figure 4D–F).

### 3.4 ELP2‐induced pyroptosis severely inhibits osteogenic differentiation

**FIGURE 5:**
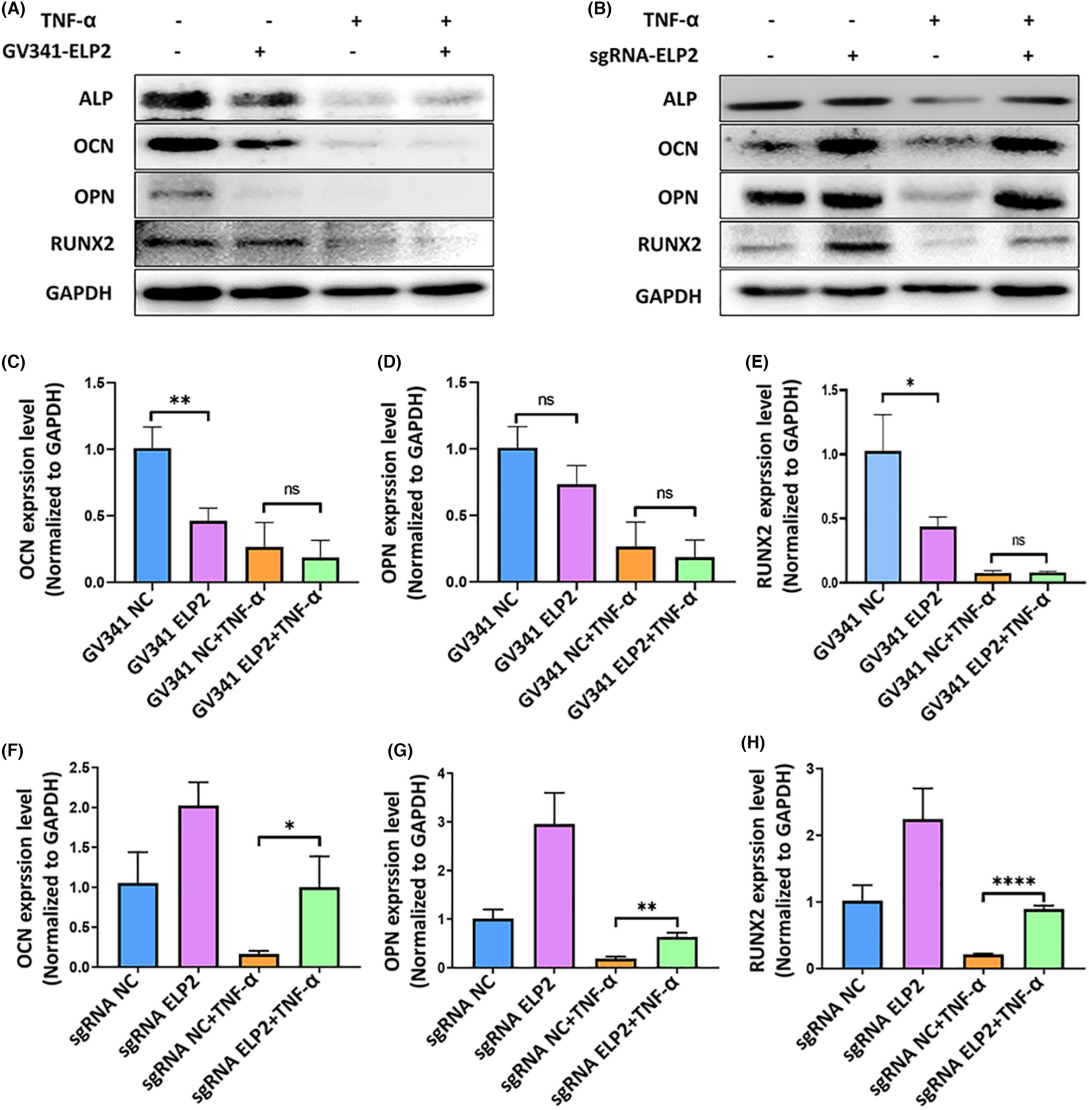
Effects of ELP2‐induced pyroptosis on osteogenesis related protein and mRNA expression. (A) Western blot analysis showed that ELP2 overexpression (GV341‐ELP2) significantly increased the reduction in OCN, OPN, Runx2 and ALP protein induced by TNF‐α (50 ng/mL 24 h); (B) ELP2 knock down (sgRNA‐ELP2) increased the reduction in OCN, OPN, Runx2 and ALP protein levels induced by TNF‐α (50 ng/mL 24 h). (C–E) Quantitative real‐time PCR analysis showed that ELP2 overexpression (GV341‐ELP2) significantly increased the reduction in OCN, OPN, Runx2, and ALP mRNA induced by TNF‐α (50 ng/mL 24 h); (F–H) ELP2 knock down (sgRNA‐ELP2) reduced the reduction in OCN, OPN, Runx2 and ALP mRNA induced by TNF‐α (50 ng/mL 24 h). Error bars represent the mean ± SD, and comparisons were performed using one‐way anova. *p > 0.05, *p < 0.05, **p < 0.01 and ***p < 0.001. *n* = 3 in each group. TNF‐α: tumour necrosis factorα; ELP2: elongator complex protein; sgRNA, small guide RNA; ALP, alkaline phosphatase; OPN, osteopontin; OCN, osteocalcin; RUNX2, Runt‐related transcription factor 2; SD, standard deviation.

Finally, we measured the protein and mRNA expression of osteogenesis‐related genes to determine whether TNF‐α leads to inflammation via the ELP2‐STAT3 pathway to inhibition new bone formation. TNF‐α exposure for 24 h significantly decreased ALP, OCN, OPN and RUNX2 protein expression compared to NC cells (Figure 5A,B), as well as OPN, OCN and RUNX2 mRNA expression (*p* < 0.05; Figure 5E–H). However, ALP, OCN, OPN and RUNX2 protein levels and OCN, OPN and RUNX2 mRNA expression all decreased to varying degrees after ELP2 overexpression, especially with TNF‐α stimulation (Figure 5A,C,D,E). Meanwhile, ELP2 knockdown significantly increased ALP, OCN, OPN, and RUNX2 protein levels and OPN, OCN, and RUNX2 mRNA levels compared to NC cells (Figure 5F–H).

**FIGURE 6:**
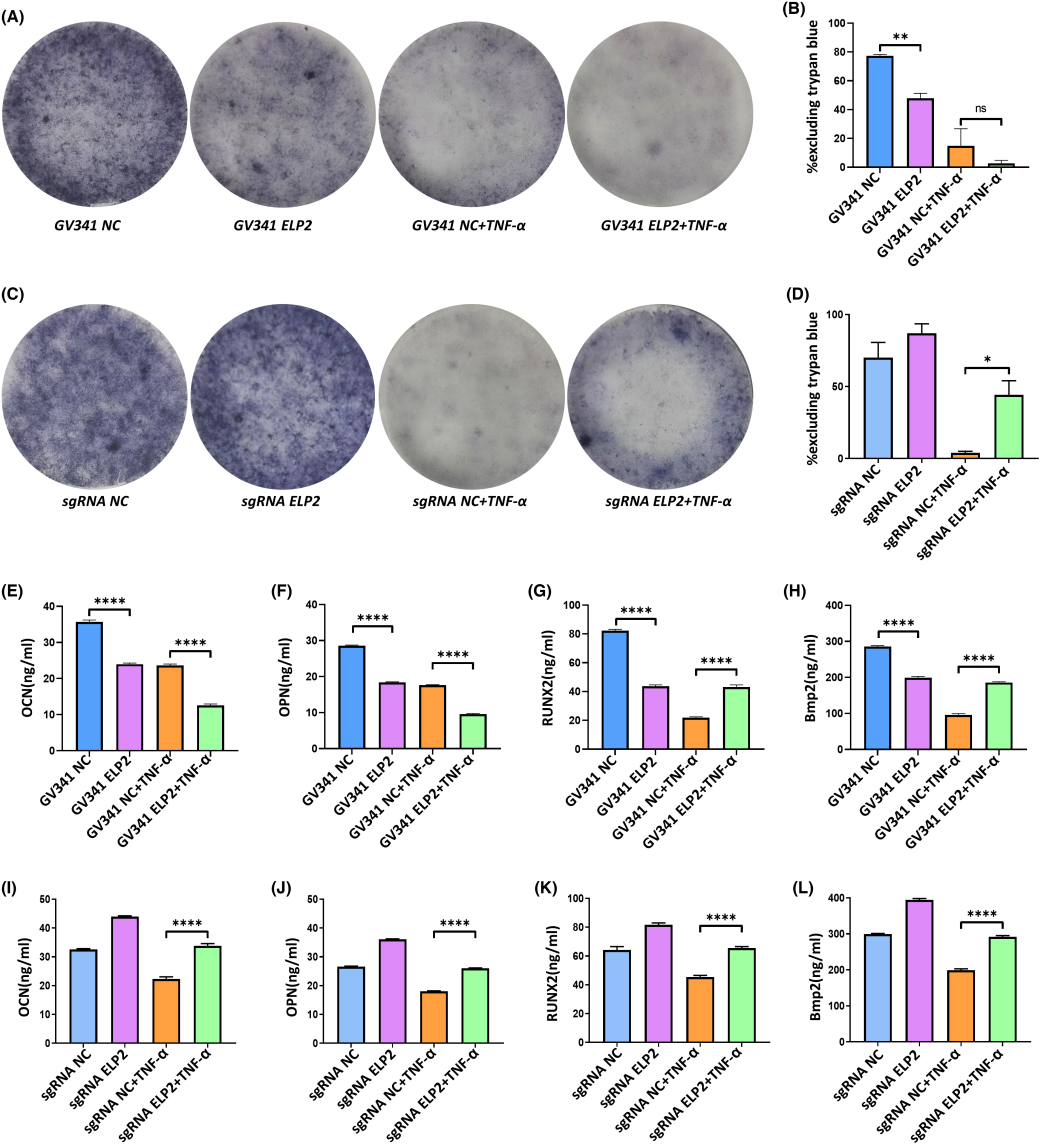
ELP2‐induced pyroptosis severely inhibits osteogenic differentiation. (A, B) ALP staining analysis showed that ELP2 overexpression (GV341‐ELP2) significantly increased the reduction in ALP level induced by TNF‐α (50 ng/mL 24 h); (C, D) ELP2 knock down (sgRNA‐ELP2) reduced the reduction in ALP level induced by TNF‐α (50 ng/mL 24 h). (E, F, G, H) ELISA analysis results showed that ELP2 overexpression (GV341‐ELP2) resulted in the significant reduction of OCN, OPN, RUNX2, and BMP2 expression, induced by TNF‐α (50 ng/mL; 24 h). (I, J, K, L) ELP2 knockdown (sgRNA‐ELP2) inhibited OCN, OPN, RUNX2, and BMP2 downregulation, induced by TNF‐α (50 ng/mL; 24 h). Error bars represent the mean ± SD, and comparisons were performed using one‐way anova. *p > 0.05, *p < 0.05, **p < 0.01 and ***p < 0.001. *n* = 3 in each group. TNF‐α: tumour necrosis factorα; ELP2, elongator complex protein; sgRNA, small guide RNA; ALP, alkaline phosphatase; SD, standard deviation.

Differences in mineralization were observed at the end of the 14‐day culture period in differentiated MC3T3‐E1 knockdown or overexpression cell cultures. TNF‐α (50 ng/mL, 24 h) seriously inhibited ALP activity, differentiation, and mineral layer formation in GV341‐NC marrow stromal cultures, as revealed by ALP staining on Day 14. Differentiation and mineral layer formation were completely inhibited when ELP2‐overexpressing MC3T3‐E1 cells were stimulated with TNF‐α (Figure 6A,C); however, ALP activity (Figure 6A,B), differentiation, and mineral layer formation (Figure 6C,D) returned to control levels following ELP2 knockdown (*p* < 0.05; Figure 6). The role of ELP2 was further verified by measuring the amount of bone‐related cytokines, such as OCN, OPN, RUNX2 and BMP2, in the supernatant using ELISAs (Figure 6E,F). Thus, ELP2 may cause severe pyroptosis, resulting in abnormal osteoblast differentiation.

## 4 DISCUSSION

In this study, we found that ELP2 is highly expressed in MC3T3‐E1 cells stimulated with TNF‐α and that ELP2 knockdown dramatically suppresses pyroptosis by inhibiting the NLRP3‐GSDMD and NLRP3‐GSDME pathways. These findings highlight pyroptosis as a biological mechanism for the pro‐inflammatory inhibition of osteogenesis and improve our understanding of the role of ELP2 and NLRP3 inflammasomes in promoting inflammatory responses during MC3T3‐E1 cell pyroptosis and osteogenesis‐related processes.

Cellular pyroptosis in inflammation is caused by TNF‐α. Therefore, preventing pyroptosis could be an attractive method for treating inflammation‐related diseases.^31^ Here, light microscopy and SEM revealed that TNF‐α induces pyroptosis. Since GSDMD and GSDME proteins form pores on the cell membrane that result in the release of inflammatory factors and dead cells subsequently aggravate inflammation, these proteins indicate the onset of pyroptosis. Since low doses of TNF‐α may induce osteogenesis, whereas high doses completely inhibit osteogenic differentiation, we established a model of osteoblast pyroptosis using high doses of TNF‐α (50 ng/mL). Our previous study suggested that the inhibitory effect of TNF‐α on osteogenesis may be associated with high expression.^8^, ^32^ Further, we conducted knock down or overexpression experiments targeting ELP2 and examined the resulting morphological changes in cells death upon stimulation with TNF‐α. We observed that TNF‐α induced death in MC3T3‐E1 cells mainly by upregulating ELP2.

Activation of NLRP3 inflammatory vesicles is thought to be an important part of the inflammatory process, often accompanied by the release of inflammatory factors. The relationship between NLRP3 and ELP2 remain unclear. Previously, we found that TNF‐α inhibits osteogenic differentiation by activating ELP2 and revealed that ELP2 and STAT3 is highly correlated with NLRP3 through bioinformatics analysis, protein docking model and co‐immunoprecipitation assays.^8^ Our study validated the hypothesis that ELP2 plays a major role in activating the STAT3‐NLRP3 inflammasome pathway and inducing pyroptosis. The protein expression of STAT3 and NLRP3 inflammasome components is increased by ELP2 overexpression, and knocking down ELP2 alleviates the increase in STAT3 and NLRP3 inflammasomes caused by TNF‐α. The simultaneous activation of NLRP3 and ASC suggests that ELP2 may play a key role by activating NLRP3 inflammasome, causing IL‐1β and IL‐18 to mature and be released extracellularly. The increase in IL‐1β and IL‐18 levels suggests that ELP2 effectively activates the cellular inflammatory environment through activation of NLRP3 inflammasome.

**FIGURE 7:**
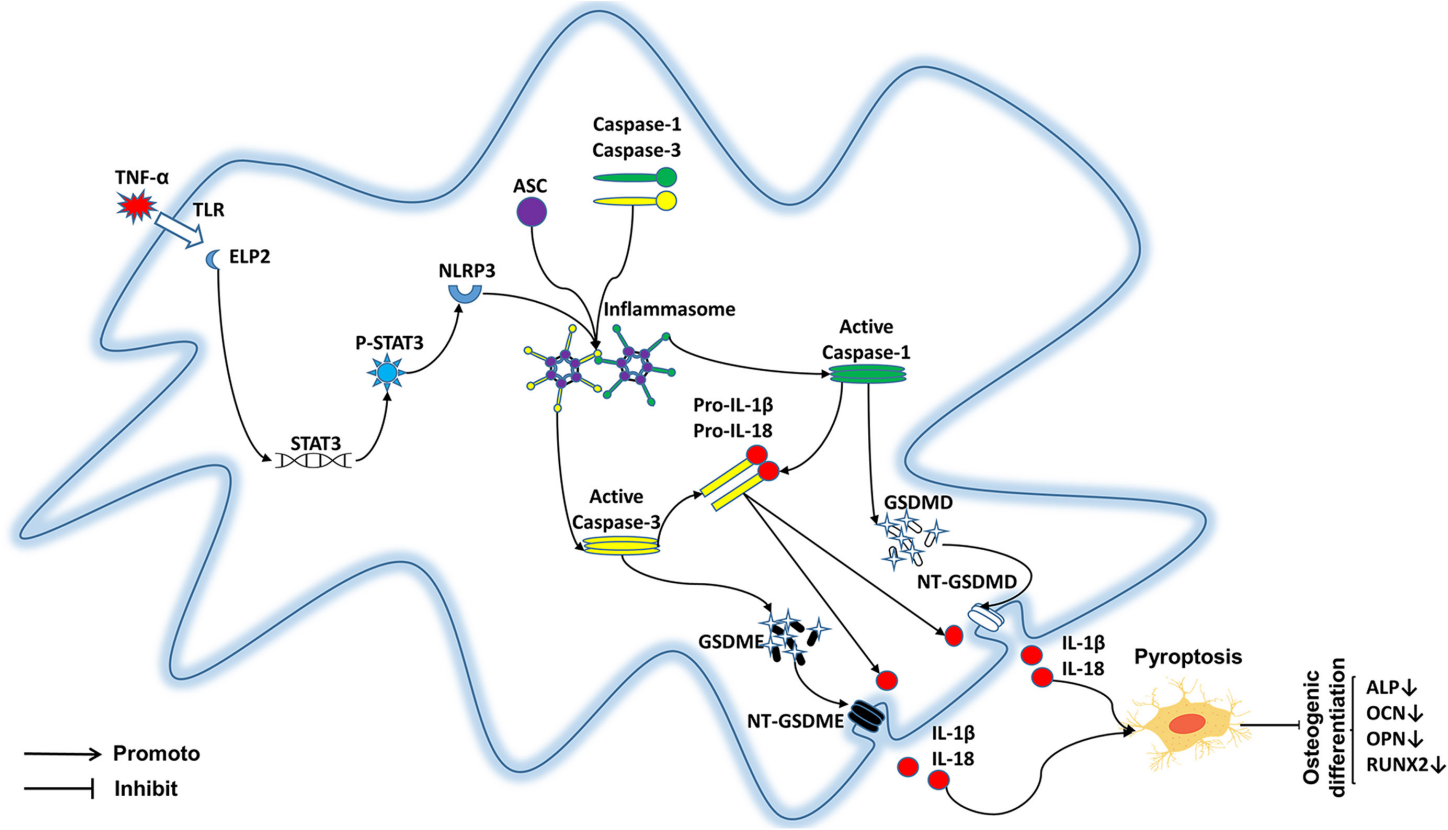
Proposed mechanical models of TNF‐α induced pyroptosis through the ELP2‐NLRP3‐GSDMD/GSDME pathway. TNF‐α leads to inflammation‐induced pyroptosis via the ELP2‐NLRP3‐GSDMD/GSDME pathway and partially underlies the adverse effects of inflammation on osteoblast differentiation. When ELP2 is upregulated STAT3 is phosphorylated. Phosphorylated STAT3 upregulated NLRP3 inflammasome causing GSDMD/GSDME cleavage, then MC3T3‐E1 cells undergo pyroptosis, osteogenic differentiation is inhibited. TNF‐α, tumour necrosis factorα; ELP2, elongator complex protein; sgRNA, small guide RNA; STAT3, signal tran sducer and activator of transcription 3; NLRP3, NOD‐like receptor thermal protein domain associated protein 3; GSDMD, Gasdermin D; GSMDE, Gasdermin E; SD, standard deviation.

Pyroptosis is a form of regulated cell death mediated by gasdermin family proteins. Osteoblasts express GSDMD and GSDME,^33^ which are associated with NLRP3 inflammasome activation and can activate pyroptosis. Notably, ELP2 overexpression can cause MC3T3‐E1 pyroptosis via the classical NLRP3 inflammasome pathway since it activates NLRP3, ASC, and caspase‐1 after 24 h of TNF‐α exposure, and causes caspase‐1 protein cleavage.^34^, ^35^ Caspase‐3 promotes GSDME pore formation during apoptosis^36^ and an increasing number of studies have suggested that caspase‐3 is highly related to NLRP3 inflammasome activation and GSDME‐induced pyroptosis.^37^, ^38^ Although the specific relationship between caspase‐3 and the NLRP3 inflammasome has not yet been elucidated, our findings suggest an association between these proteins. We speculate that caspase‐3, like caspase‐1, can activate the NLRP3 inflammasome, possibly through competitive inhibition. In addition, we found that caspase‐3 overexpression is not conducive to GSDMD cleavage by caspase‐1 ^14,33^ (Figure 7). This phenomenon suggests that blocking only caspase‐1 or caspase‐3 does not effectively inhibit pyroptosis. The possible competitive inhibitory relationship between caspase‐1 and caspase‐3 in pyroptosis is worth further exploration. Determining the role of caspase‐1 and caspase‐3 may hold promise for developing novel therapeutic strategies to mitigate the detrimental effects of excessive inflammation in various human diseases.

ALP, OCN, OPN and RUNX2 play an important role in the regulation of osteogenic differentiation of bone calcium content and regulation of bone calcium metabolism.^39^ In general, changes in levels through ALP, OCN, OPN, and RUNX2 are considered important indicators to reflect osteogenesis. In particular, the degree of ALP staining is significantly reduced after being stimulated by TNF‐α or overexpressing ELP2, and the downward regulation of ALP levels caused by TNF‐α can be alleviated by knocking down ELP2. Therefore, TNF‐α causes cell pyroptosis through the ELP2‐NLRP3 pathway, resulting in a decrease in the number of cells and inhibiting the occurrence of osteogenic differentiation.

Despite these findings, our study has some limitations. For instance, only TNF‐α was used as an inflammatory stimulus and only MC3T3‐E1 was used as a representative of osteoblasts; the relationship between caspase‐3 and NLRP3 inflammasomes has not yet been fully clarified and there are no good blocking agents that can block GSDME. Finally, we only used SEM and analysed pyroptosis proteins (GSDMD/GSDME) to confirm the occurrence of pyroptosis. The use of immunofluorescence or transmission electron microscopy can further validate the obtained results.

The identification of gasdermin family members as the final effectors of pyroptosis and the release of inflammatory cytokines following inflammasome activation has suggested a new strategy for treating inflammatory diseases.^40^ Here, we found that ELP2‐induced pyroptosis severely inhibits osteoblast differentiation, either through GSDME or GSDMD. Therefore, inhibiting ELP2 can reduce inflammation by limiting the activation of inflammasome pathways, making the inhibition of pyroptosis an attractive therapeutic option.

## 5 CONCLUSION

Taken together, our data indicate that ELP2 activation creates an inflammatory microenvironment and leads to pyroptosis through the NLRP3 inflammasome pathway. In particular, ELP2 promotes both the caspase‐1/GSDMD and caspase‐3/GSDME pathways, which regulate NLRP3 inflammasomes and lead to vascular endothelial cell pyroptosis that aggravates inflammation and affects osteogenic differentiation.

## AUTHOR CONTRIBUTIONS

**Chang‐liang Xia:** Conceptualization (lead); data curation (lead); formal analysis (lead); investigation (lead); methodology (lead); project administration (lead); resources (equal); validation (lead); writing – original draft (lead); writing – review and editing (lead). **Shuang‐ji Ou:** Conceptualization (supporting); data curation (supporting); formal analysis (supporting); methodology (supporting); resources (supporting); supervision (supporting). **Yang Yang:** Conceptualization (supporting); data curation (supporting); funding acquisition (supporting); methodology (supporting); supervision (supporting); visualization (supporting); writing – original draft (supporting). **Wei Zhang:** Formal analysis (supporting); investigation (supporting); project administration (supporting). **Wen‐jiao Wu:** Conceptualization (supporting); data curation (supporting); visualization (supporting). **Qi Chen:** Formal analysis (supporting); funding acquisition (supporting); methodology (supporting); resources (supporting); supervision (supporting); writing – original draft (supporting). **Wen‐jun Li:** Data curation (supporting); methodology (supporting); validation (supporting). **Han‐yu Lu:** Conceptualization (supporting); investigation (supporting); visualization (supporting). **Ye‐yang Wang:** Conceptualization (supporting); methodology (supporting); project administration (supporting). **Yong Qi:** Conceptualization (equal); data curation (equal); formal analysis (equal); funding acquisition (equal); methodology (equal); supervision (equal); validation (equal); visualization (equal); writing – review and editing (equal). **Changpeng Xu:** Conceptualization (supporting); data curation (supporting); funding acquisition (supporting); resources (supporting).

## FUNDING INFORMATION

This work was supported by the National Natural Science Foundation of China (No. 81972083), the Science and Technology Planning Project of Guangzhou (No. 202201020303, 202102080052, 202102010057, 201804010226), and the Foundation of the Guangdong Second Provincial General Hospital (No. 3D‐A2020004, 3D‐A2020002, YQ2019‐009, C2020019).

## CONFLICT OF INTEREST STATEMENT

The author reports no conflicts of interest in this work.

## Data Availability

The datasets GENERATED for this study can be found in the Targetscan (https://www.targetscan.org/vert_80/).
